# Antimicrobial use in Swedish farrow-to-finish pig herds is related to farmer characteristics

**DOI:** 10.1186/s40813-016-0035-0

**Published:** 2016-08-01

**Authors:** Annette Backhans, Marie Sjölund, Ann Lindberg, Ulf Emanuelson

**Affiliations:** 1 Department of Clinical Sciences, Swedish University of Agricultural Sciences, SE-750 07 Uppsala, Sweden; 2 Department of Animal Health and Antimicrobial Strategies, National Veterinary Institute, SE-751 89 Uppsala, Sweden; 3 Department of Epidemiology and Disease Control, National Veterinary Institute, SE-751 89 Uppsala, Sweden

**Keywords:** Antimicrobial, Biosecurity, Pig, Farmer attitudes

## Abstract

**Background:**

Antimicrobial resistance is an increasing problem and reducing AM use is critical in limiting its severity. The underlying causes of antimicrobial use at pig farm level must be understood to select effective reduction measures. We previously showed that antimicrobial use on Swedish pig farms is comparatively low but varies between farms, although few farms are high users. In the present survey of a convenience sample of 60 farrow-to-finish herds in Sweden, we investigated farmers’ attitudes to antimicrobials and the influence of information provided by veterinarians about antimicrobial resistance. Farm characteristics were also recorded. We had previously quantified antimicrobial use for different age categories of pigs during one year, as well as external and internal biosecurity. Risk factors based on hypothetical causal associations between these and calculated treatment incidence (TI) for the different age categories were assessed here in a linear regression model.

**Results:**

There were no significant associations between biosecurity and TI for any pig age category. Increasing farmer age was associated with higher TI for suckling piglets and fatteners. For suckling piglets, the age group with the highest frequency of treatment, TI was also significantly associated with farmer and education of the staff, where female farmers, and university educated staff was associated with a higher TI. Larger farms were associated with a higher TI in fatteners.

**Conclusions:**

In the investigated Swedish pig farms, factors that influenced antimicrobial usage were more related to characteristics of the individual farmer and his/her staff than to biosecurity level, other management factors or farmers’ attitudes to antimicrobials.

## Background

Antimicrobial (AM) use in animal production in Sweden is among the lowest in Europe [1]. This is explained partly by absence of diseases such as porcine reproductive and respiratory syndrome (PRRS) together with a long tradition of implementing preventative measures against livestock diseases [2] and a ban on the use of AM as growth promoters since 1986, which decreased AM use by 65 % [3]. Within the European research project MINAPIG, we recently showed that AM use in Swedish pig herds mainly consists of individual treatments and that most herds apply AM prudently, with rather low use of fluoroquinolones and no use of third-generation cephalosporins [4]. Investigation of the biosecurity level in the same herds showed that, in general, the biosecurity was good, but varied between herds [5]. Thus, Swedish pig production has come a long way in reducing AM use, but the great variation between farms indicates that some farms could reduce use even further.

The presence of infectious diseases in an area has an impact on the health status of pigs, but various biosecurity measures can be applied to prevent pathogens entering or spreading within a herd, thereby improving animal health [6–9]. Thus, improvements in biosecurity could be useful to reduce the need for AM in pig herds. Furthermore, the process by which the farmer decides how to apply treatments has been shown to be influenced by their attitudes and beliefs regarding antimicrobials [10]. Previous studies have shown that farmers generally have little awareness of the risks of AM resistance [11–13], and that they are more concerned about financial issues [14, 15]. To date, very few studies have taken into account both preventive measures and attitudes to AM use [16].

Our recent study showed that the level of AM use varies greatly between pig herds and that there is room for improvement, especially with regard to treatments in suckling piglets [4]. Therefore, the aim of the present study was to investigate the farm, or farmer-related, factors influencing AM use on Swedish farrow-to finish pig farms, and how biosecurity level, farmers’ attitudes to AM and the information provided by the herd veterinarian influence AM use under Swedish conditions. The hypotheses tested were: that a high level of biosecurity is associated with lower AM use; that farmers who are aware of the risks of AM resistance use less AM; and that information provided by veterinarians has an impact on AM use.

## Methods

### Herds and collection of data

The study was performed within the European research project MINAPIG (Evaluation of alternative strategies for raising pigs with minimal antimicrobial usage). The study design has been described in detail in our previous publications [4, 5]. In brief, 60 Swedish farrow-to-finish herds, with at least 100 sows and 500 finishing pigs per year, were recruited by convenience sampling. The selection criteria were agreed within the MINAPIG project to ensure comparable samples between the participating countries. The herds were visited once during the period April-September 2013, when data on production parameters, biosecurity practices and other herd characteristics were collected by a researcher or the herd veterinarian. A questionnaire on farmers’ perceptions on AM use, previously described by Visschers et al. 2015 [15] was filled out before the visit by the person responsible for pig management and collected together with records of the amount of AM used during the year preceding the visit.

### Calculation of antimicrobial use

Use of AM was recorded by product, strength of product, administration route and age category. The values were converted to active substance, expressed as mg, and then to treatment incidence (TI) based on Defined Daily Doses for Animal (DDDA) previously agreed within the MINAPIG project [17]. This was done using the online tool ABcheck (available at www.ABcheck.ugent.be), but adapted to the MINAPIG project (www.minapig.eu). The TI was expressed as the number of DDDAs per 1000 pig-days at risk, which is equivalent to the proportion of 1000 pigs that receive a dose of AM each day [18]. The TI values were calculated separately for suckling piglets (birth to weaning), weaners (weaning to an approximate weight of 30 kg), fatteners (~30 kg to slaughter) and adult pigs (gilts, sows and boars). Further details about these calculations can be found in our previous publication [4].

### Assessment of biosecurity

Biosecurity practices applied in the herds were evaluated using the online tool BioCheck (available at www.biocheck.ugent.be) developed by Laanen et al. 2010 [19] and modified for MINAPIG. In brief, BioCheck consists of 109 questions relating to biosecurity measures, grouped into 6 subcategories of each of external and internal biosecurity measures. Examples of external biosecurity measures are “Purchase of animals and semen” and “Transport of animals and removal of manure and dead animals”, and of internal biosecurity measures “Disease management”, “Biosecurity measures between compartments and the use of equipment” and “Cleaning and disinfection”. The score for each subcategory accounts for its estimated importance for the introduction and spread of infectious diseases, with scores ranging between 0, corresponding to “total absence of biosecurity” and 100, corresponding to “perfect biosecurity”.

### Farmers’ attitudes to antimicrobial use and the influence of veterinarians

A questionnaire was developed within the MINAPIG consortium based on semi-structured interviews with 14 pig farmers in Switzerland and Germany (for details see Visschers et al. 2015 [15]). The questionnaire was developed in English, but subsequently translated to Swedish and distributed before the farm visit with a request that it be filled out by the farmer or the person responsible for the pigs (hereafter referred to as ‘the farmer’). The questionnaire contained questions about age, gender and years of experience. It also included statements on the benefits and risks of AM use in pig farming, the need to apply AM in pig farming and the information provided by the farm veterinarian regarding AM use. The statements were assessed on a 6-point Likert scale, where higher scores indicated stronger agreement with the respective item, and further combined into four constructs: perceived benefits, perceived risks, perceived need and contribution from veterinarians, each based on a number of individual items. These constructs have been described previously [15, 16] and are presented in Table 1. As all constructs had acceptable to good internal reliability (Cronbach’s alpha values between 0.64 and 0.83), the mean for these items per respondent was calculated and used for the constructs in the analyses.

**Table 1:** Statements included in the constructs related to farmers’ attitudes to antimicrobial use and to the influence of veterinarians

Construct	Statements	No of answers per score of each statement: 1 (do not agree at al), 6 (fully agree)					
		1	2	3	4	5	6
Perceived risks	AB are associated with risks for the pigs	11	18	13	10	5	2
	AB use in pig farming reduces the effectiveness of ABs in human medicine	5	12	14	11	8	9
	AB are used far too much in pig production	10	15	11	13	5	5
Perceived benefits	AB can be easily and quickly applied	3	5	13	7	15	15
	AB are very cost efficient	3	3	16	10	17	10
	The effect of AB in pigs is very fast	0	0	13	18	14	14
	The animals recover quickly due to AB	0	1	8	16	21	13
	AB highly reduce the number of deaths among pigs	2	11	8	13	15	10
Perceived need of AM	Keeping a large number of pigs is only possible with the intensive use of AB	19	23	6	8	2	1
	Disease incidents caused by the conditions of intensive pig farming can only be cured by AB	23	11	9	8	6	2
Contribution from veterinarians	My veterinarian informs me about the risks of antibiotic use	1	2	3	4	15	33
	My veterinarian informs me about how AB work	0	1	4	5	20	28
	My veterinarian informs me about the impact of alternative strategies and how to use them	1	1	9	11	16	21

*AB* antibiotic

### Statistical analyses

**Fig. 1:**
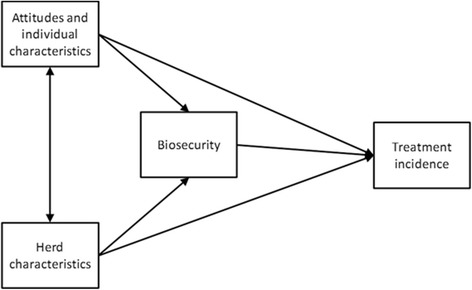
A directed acyclic graph illustrating the hypothetical causal associations between risk factors (shown as groups) and antimicrobial treatment incidence in Swedish farrow-to-finish herds

A directed acyclic graph (DAG) illustrating the hypothetical causal associations between assumed risk factors and frequency of AM use (expressed as TI) is shown in Fig. 1. Herd characteristics considered for the linear regression models were: number of sows, number of employees, whether the farm was specific pathogen free (SPF) or not, and average reported age at weaning. Individual characteristics were age, gender and years of experience of the farmer, and highest level of education of the staff. Attitudes considered important were the four constructs (perceived benefits of AM, perceived risks of AM, perceived need for AM and information contribution from veterinarians). All candidate risk factors, except gender, level of education and SPF status, were measured on a continuous scale and the assumption of linear associations with the outcome was managed by introducing a quadratic term after centring on the mean, which was retained in the model if it was statistically significant (*p* < 0.05). Multicollinearity between the potential predictor variables was assessed by Spearman rank correlations. SPF status was found to be highly correlated with external biosecurity and was excluded from the model, because external biosecurity was better distributed and was of primary interest in this study. Number of employees was highly correlated with number of sows and only the latter was retained in the regression models. All TI values were log-transformed (natural base) to achieve normally distributed residuals, where 1 was added to all TI values for weaners, fatteners and adults to avoid taking the log of zero.

## Results

### Farm and farmer characteristics, farmers’ attitudes, veterinarians’ information contribution, AM use and biosecurity level

Descriptive statistics on herd and individual characteristics, including attitudes to AM and the contribution of veterinarians, are presented in Tables 1 and 2. Three herds were SPF herds, gender distribution was 18 females and 41 males (one farmer did not indicate their gender) and the level of education was 20 farmers with a university degree and 40 without. There was great variation in number of sows, age and years of experience of the farmer and weaning age. Farmers perceived a low need, low-moderate risks and moderate benefits of AM and rated the contribution of their veterinarian’s information highly.

**Table 2:** Characteristics of the 60 Swedish farrow-to-finish pig herds surveyed

	Mean	Minimum	25th percentile	Median	75th percentile	Maximum
Number of sows	242.7	96	137.5	187.5	275	1200
Number of employees	3.9	2	3	3	4	15
Farmer age	49.7	27	44	49	58	70
Years of experience	22.3	3	15	22	28	50
Pig age at weaning (days)	35	28	33	35	35	49
Total duration (weeks)^a^	26.9	23	25.6	27	28	32
Perceived need for AM^b^	2.3	1	1	2	3	6
Perceived risks of AM^b^	3.1	1	2.3	3	3.8	6
Perceived benefits of AM^b^	4.3	1.8	3.6	4.2	5	6
Veterinarians’ information contribution^b^	5	1	4.5	5	6	6
Internal biosecurity^c^	58.8	33	52	61	65.5	80
External biosecurity^c^	68.3	44	61.5	68	76	93

^a^Total duration = entire rearing period from birth to slaughter, data available for 51 herds, AM = Antimicrobial; ^b^The original items were assessed on a 6-point Likert scale, where higher scores indicated stronger agreement with the respective item; ^c^Scores for internal and external biosecurity range between 0 and 100, where 100 is “perfect biosecurity”

The distribution of internal, external and total biosecurity for the participating herds is also presented in Table 2 and the distribution of TI values for the different pig age categories is shown in Table 3.

**Table 3:** Distribution of antimicrobial use for different age groups of pigs, expressed as treatment incidence per 1000 pig-days at risk, in 60 Swedish farrow-to-finish herds

	Mean	Minimum	25th percentile	Median	75th percentile	Maximum
Suckling piglets	75.1	1.6	21.1	54.7	103.2	367.9
Wearers	22.3	0.0	2.1	6.2	20.1	260.5
Fatteners	6.1	0.0	1.6	2.8	6.1	64.9
Adults	10.9	0.0	4.2	8.4	15.4	45.0

These data, including detailed information on use of AM substances and biosecurity scores for subcategories, have been published in our two previous papers [4, 5]. As reported there, the TI was highest for suckling piglets and second highest for weaners [4]. However, the TI varied greatly between farms, especially for suckling piglets and weaners. The external biosecurity was higher than the internal biosecurity, but there were few herds with low external biosecurity and few with perfect external biosecurity [5].

### Regression analysis

In the regression models including only internal and external biosecurity (results not shown), there were significant associations between internal biosecurity and TI in weaners, fatteners and adults, and between external biosecurity and TI in weaners. However, the linear regression models that also included herd characteristics and individual characteristics and attitudes showed no significant associations between biosecurity and TI for any of the pig age groups (Table 4). The factor most consistently associated with TI was the farmer’s age, with higher age being associated with higher TI in suckling piglets and fatteners. In suckling piglets, the age group to which most treatments were applied, TI was significantly associated with age, gender and education, with higher age, female farmer and university education of the farmer being associated with higher TI. Large farm size, indicated by number of sows, was associated with higher TI in fatteners. The fit of the models was assessed by inspection of the residuals with respect to homoscedasticity and normal distribution, but no deviations were found.

**Table 4:** Estimates from a linear regression model of the associations between risk factors and antimicrobial (AM) use (expressed as log-transformed (natural base) treatment incidence (TI) per 1000 pig-days at risk) in different age groups of pigs on 60 Swedish farrow-to-finish herds

	Suckling piglets			Weaners			Fatteners			Adults		
Risk factor	Estimate	Error	*p*	Estimate	Error	*p*	Estimate	Error	*P*	Estimate	Error	*p*
Intercept	-2.385	3.695		-4.561	4.980		0.569	2.205		3.338	2.138	
Number of sows	0.000	0.001	0.975	0.000	0.001	0.881	0.005	0.003	0.060	0.001	0.001	0.319
Number of sows^2*^							0.000	0.000	0.042			
Gender - male (*n = 41*)	-0.877	0.370	0.018	-0.447	0.370	0.226	-0.178	0.370	0.631	-0.003	0.370	0.995
- female *(n = 18)*	0.000	0.000		0.000	0.000		0.000	0.000		0.000	0.000	
Education - non-uni^a^ *(n = 39)*	-0.725	0.323	0.025	-0.464	0.324	0.152	-0.112	0.331	0.736	-0.159	0.322	0.622
- uni *(n = 20)*	0.000	0.000		0.000	0.000		0.000	0.000		0.000	0.000	
Age	0.392	0.131	0.003	0.351	0.146	0.016	0.021	0.027	0.439	-0.040	0.026	0.122
Age^2*^	-0.004	0.001	<0.001	-0.004	0.002	0.013						
Years at work	0.027	0.027	0.310	-0.005	0.028	0.867	-0.016	0.027	0.562	0.014	0.027	0.600
Age at weaning	^b^na			0.032	0.043	0.466						
Need for AM^c^	0.092	0.126	0.4682	-0.047	0.126	0.712	0.044	0.126	0.726	0.028	0.126	0.823
Risks of AM^c^	-0.196	0.143	0.1695	-0.210	0.145	0.148	0.052	0.143	0.719	-0.001	0.143	0.994
Benefits of AM^c^	-0.119	0.155	0.442	0.233	0.155	0.132	0.286	0.156	0.068	0.233	0.154	0.130
Vet’s contribution^c^	0.121	0.135	0.372	-0.003	0.135	0.984	-0.134	0.152	0.378	0.141	0.135	0.297
Internal biosecurity^d^	-0.022	0.019	0.237	0.004	0.019	0.823	-0.031	0.019	0.095	-0.024	0.018	0.192
External biosecurity^d^	-0.002	0.021	0.913	-0.033	0.021	0.115	0.011	0.020	0.578	0.001	0.020	0.957

* The quadratic term a*uni* university, *vet* veterinarian, ^b^ *na* not applicable; ^c^Statements were assessed on a 6-point Likert scale, where higher scores indicated higher agreement, combined into four constructs and expressed as the mean score per construct; ^d^Scores for internal and external biosecurity range between 0 and 100, where 100 is “perfect biosecurity”

## Discussion

Use of AM is lower in pig production in Sweden than in most other European countries [20], but we previously showed that the level varies between farms, with a few farms being high users [4, 5]. The biosecurity level also varies greatly between farms, as do other characteristics such as age, years of experience and education level of the farmer. In this study, two of our starting hypotheses were that a high level of biosecurity is associated with lower AM use (as a result of better pig health), and that farmers who are aware of the risks of AM resistance use less AM. However, the results from the linear regression model showed that the associations between AM use and biosecurity were non-significant when farm and farmer characteristics were included in the model. Furthermore, attitudes to AM were not significantly associated with AM use. Instead, individual characteristics of the farmer were found to be important. For example, older farmers, females and university-educated farmers (any university education) used more AM in suckling piglets, which is the age group to which most treatments are applied [4], and older farmers also used more AM in weaners. We previously reported a link between fewer years of farmer experience, female farmers and higher biosecurity [5], which led us to expect lower AM use on such farms, but surprisingly gender affected AM use in a different way than expected. Moreover, it could be assumed that a high level of education would lead to more careful use of AM, not more frequent use as found here. However, as discussed in our previous paper [5], females have been shown to have generally higher empathy for animals [21] but also higher medical compliance than men, who tend to show riskier behaviour relating to health issues [22]. The results could therefore be due to females’ greater attention to the individual animal’s symptoms. The reason why older and more educated farmers had higher AM use than younger and less educated farmers can only be speculated upon, but might be due to similar factors. It could also be speculated whether this means there is over-treatment, especially of suckling piglets, in some herds, or under-treatment in others. The health status in Swedish pig production is generally good, but examples of diseases that are prevalent and often need treatment are arthritis and neonatal piglet diarrhoea in suckling piglets, diarrhoea in weaners, respiratory diseases in fatteners and udder- and leg-related diseases in sows [23–25].

The lack of association between AM use and biosecurity in the present study was unexpected, especially because a negative association between biosecurity level and estimated frequency of treatment for certain clinical signs of disease was reported in a parallel study across four countries [26]. One explanation could be that some of the Swedish herds with otherwise good pig health might have experienced an outbreak of disease, leading to temporarily high AM use. Moreover, herds struggling with health problems might have implemented biosecurity measures to overcome the problem, affecting the results in this limited sample of herds. Both these explanations could result in lack of an association. Furthermore, the most important biosecurity measures, such as all-in all-out systems or lower stocking density than the EU limit [5], might have been implemented already in the majority of our herds. Swedish herds have in general better biosecurity level than herds in other countries [26] and associations with AM use may thus be more difficult to identify. It is also possible that BioCheck, a tool developed in Belgium, is not entirely appropriate for Swedish conditions. The prevalence of infectious diseases differ between the countries and Sweden is for example declared free from PRRS [2]. Also, pig density is overall considerably lower in Sweden [27]. Consequently, the assumption that a certain biosecurity level (as measured by BioCheck) has the same effect on AM needs may not be valid.

Finally, the associations in the final model, i.e. after accounting for other factors such as herd and farmer characteristics, were marginal, which indicates that biosecurity level is a less important determinant of AM use in Swedish pig herds, perhaps due to an overall better health status. However, it cannot be excluded that the sample size of 60 herds was not sufficient to detect associations when several risk factors were included in the model and the absence of statistically significant associations should not be interpreted as a proof that there is no association. Greater farm size, defined as number of sows, was a significant factor for higher AM use in fatteners, but not in other age groups. Several studies have identified increasing herd size as a risk factor for respiratory disease, which is mainly a problem during the fattening period [28, 29]. The lack of associations with farmers’ perceived risks, benefits and need for AM could be due to the relatively narrow distribution of scores, i.e. farmers’ attitudes were too similar to be able to identify any differences. Moreover, scores for the information contribution from veterinarian construct did not differ much between herds. Thus, it cannot be concluded that attitudes are unimportant in explaining AM use, but the results indicate that there is consensus among farmers on their attitudes to AM, perhaps influenced by the issue being a topic debated in society and within pig production. In Sweden, veterinarians are not allowed to sell AM and prescriptions are restricted to named AM products in quantities the veterinarian considers necessary during a limited period, based on regular monitoring of the health status of the pigs and AM use. Moreover, the farmer must undergo special training to administer treatments [30, 31]. These regulations are likely to contribute to awareness about the risks of AM use.

Limitations of the present study to consider are that AM use in the participating herds was lower compared to national AM sales figures for the same period [4], indicating a bias towards farms with lower AM use than the average pig farm. Further, about one third of Swedish herds are farrow-to-finish herds and the study group, a convenience sample of Swedish medium-sized and large herds, represented approximately 22 % of farrow-to-finish herds with >100 sows. Thus actual high users might not have been very well represented in our sample and it is possible that these farmers have different views on AM and their herds have lower biosecurity.

## Conclusions

Factors influencing AM use in Swedish farrow-to-finish pig farms were related to individual farmer characteristics such as age, gender and years of experience. However, under Swedish circumstances, biosecurity level had no additional effect on AM use. This indicates the importance of the herd veterinarian’s communication skills to ensure correct treatment of sick animals.
